# Active full-length DNA Aβ_42_ immunization in 3xTg-AD mice reduces not only amyloid deposition but also tau pathology

**DOI:** 10.1186/s13195-018-0441-4

**Published:** 2018-11-20

**Authors:** Roger N. Rosenberg, Min Fu, Doris Lambracht-Washington

**Affiliations:** Department of Neurology and Neurotherapeutics, University of Texas Southwestern Medical Center Dallas, 5323 Harry Hines Boulevard, Dallas, TX 75390-8813 USA

**Keywords:** Alzheimer’s disease, Immunotherapy, DNA vaccination, Amyloid-β, Aβ oligomer, Tau, Tau kinases

## Abstract

**Background:**

Alzheimer’s disease (AD) is the most well-known and most common type of age-related dementia. Amyloid deposition and hyperphosphorylation of tau protein are both pathological hallmarks of AD. Using a triple-transgenic mouse model (3xTg-AD) that develops plaques and tangles in the brain similar to human AD, we provide evidence that active full-length DNA amyloid-β peptide 1–42 (Aβ_42_) trimer immunization leads to reduction of both amyloid and tau aggregation and accumulation.

**Methods:**

Immune responses were monitored by enzyme-linked immunosorbent assay (ELISA) (antibody production) and enzyme-linked immunospot (cellular activation, cytokine production). Brains from 20-month-old 3x Tg-AD mice that had received DNA Aβ_42_ immunotherapy were compared with brains from age- and gender-matched transgenic Aβ_42_ peptide-immunized and control mice by histology, Western blot analysis, and ELISA. Protein kinase activation and kinase levels were studied in Western blots from mouse hemibrain lysates.

**Results:**

Quantitative ELISA showed a 40% reduction of Aβ_42_ peptide and a 25–50% reduction of total tau and different phosphorylated tau molecules in the DNA Aβ_42_ trimer-immunized 3xTg-AD mice compared with nonimmunized 3xTg-AD control animals. Plaque and Aβ peptide reductions in the brain were due to the anti-Aβ antibodies generated following the immunizations. Reductions of tau were likely due to indirect actions such as less Aβ in the brain resulting in less tau kinase activation.

**Conclusions:**

The significance of these findings is that DNA Aβ_42_ trimer immunotherapy targets two major pathologies in AD—amyloid plaques and neurofibrillary tangles—in one vaccine without inducing inflammatory T-cell responses, which carry the danger of autoimmune inflammation, as found in a clinical trial using active Aβ_42_ peptide immunization in patients with AD (AN1792).

## Introduction

Immunotherapeutic approaches have high potential for successful treatment interventions in Alzheimer’s disease (AD). Following the lessons learned from the first anti-amyloid-β peptide 1–42 (anti-Aβ_42_) clinical trial (AN1792), in which patients with AD received an Aβ_42_ vaccine and QS-21 adjuvant, which led to encephalitis in 6% of the treated patients, a major focus is now on avoiding autoimmune inflammation [1–3]. Ongoing clinical trials are pursuing passive vaccination with mouse monoclonal antibodies (mAbs) or fully human antibodies against Aβ_42_ peptide epitopes to avoid complications from autoimmunity [4–7]. A recent study in which patients received passive immunotherapy with an mAb targeting oligomeric or prefibrillar Aβ_42_ reported positive results regarding amyloid reduction in the brain as well as improved cognitive measurements [8].

Besides amyloid accumulation, tau aggregation and spreading have been associated with progression of AD. In fact, increased tau levels showed high correlation with cognitive decline in patients with AD [9]. Tau immunotherapy is being evaluated in various preclinical and clinical trials as well, using active immunizations with peptides from different parts of the tau protein or passive immunizations using polyclonal or mAbs [10–15]. Antitau antibodies have been shown to act inside and outside of neurons and to reduce tau hyperphosphorylation as well as pathogenic tau seeding [16–20].

We report, for the first time in an AD mouse model, that active DNA Aβ_42_ immunization into the skin targets two pathologies: amyloid-containing plaques and tau. DNA vaccination, in which not the antigen (peptide or protein) but the DNA encoding this peptide is administered, is an alternative route of vaccination. Genes encoded by the DNA are expressed within the skin, and the peptides are taken up by dendritic cells traveling to the regional lymph nodes and presenting the antigen to B and T cells [21]. Immune responses to DNA or peptide immunization differ qualitatively. We have shown previously that full-length DNA Aβ_42_ trimer immunization is noninflammatory and induces a regulatory immune response [22–25]. DNA Aβ_42_ trimer immunization has been shown to be effective in removing amyloid from the brain in immunized double-transgenic mice (APPswe/PS1 [26–28]). In the present study, we used a triple-transgenic AD mouse model (3xTg-AD) that exhibits Aβ and tau pathologies characteristic of human AD [29, 30]. We found that immunotherapy with DNA Aβ_42_ trimer leads to reduction of Aβ_40_/Aβ_42_ peptides and amyloid plaques, and we show for the first time that DNA Aβ_42_ trimer immunization leads also to significant reduction of tau from the mouse brain.

## Methods

### Animals

3xTg-AD [B6;129-Tg(APPSwe,tauP301L)1Lfa Psen1^tm1Mpm^/Mmjax, MMRRC Stock No: 34830-JAX] mice had been purchased from the Mutant Mouse Research and Resource Center at The Jackson Laboratory and were bred and housed at the UT Southwestern Medical Center animal facility under conventional conditions. This mouse model had been developed by Oddo and colleagues [29, 30]. Animal use was approved by the UT Southwestern Medical Center Animal Research Committee, and animal research was conducted under the Animal Research: Reporting of In Vivo Experiments guidelines [31].

### Study design

Cohorts of 3xTg-AD mice were immunized with a DNA Aβ_42_ trimer vaccine, Aβ_42_ peptide (rPeptide, Watskinville, GA, luciferase (Luc) control DNA, or left untreated as controls. This mouse model that had been developed by Oddo and colleagues develops plaque and tangle pathology [29, 30]. Cohort 1 consisted of 16 Tg female mice and 8 wild-type controls, and cohort 2 consisted of 34 Tg female mice. Parallel immunized groups of 3xTg-AD males (16 males in cohort 3, 15 males in cohort 4) showed no plaque pathologies at 18 and 20 months of age, so this study used females only. The mice were vaccinated at a total of 13 time points until 20 months of age for final analyses. Collected brains were cut in a sagittal plane. One hemibrain was frozen and used in enzyme-linked immunosorbent assays (ELISAs) and Western blots, and the other half was fixed for immunostaining with anti-Aβ and antitau antibodies.

### Immunizations and collection of blood samples

**Fig. 1:**
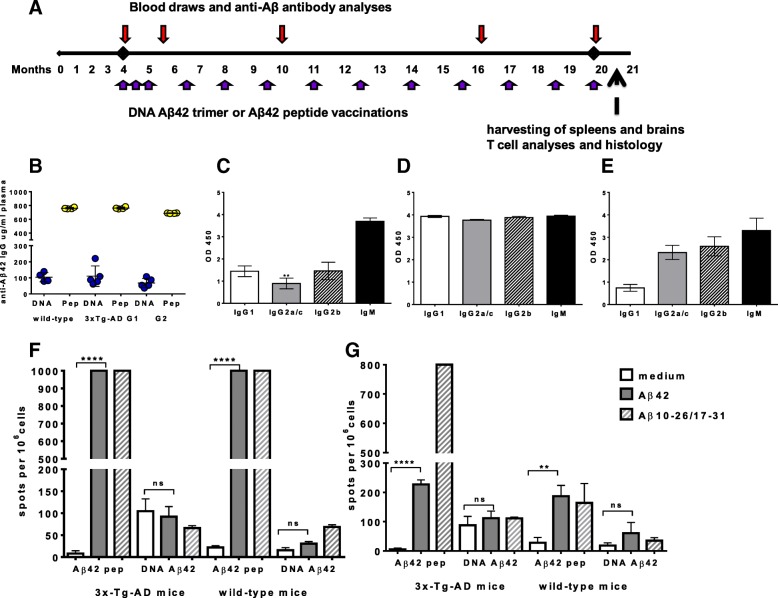
Timeline of the experimental procedures, anti-amyloid-β peptide 1–42 (anti-Aβ_42_) antibody production upon immunization with DNA Aβ_42_ trimer and Aβ_42_ peptide in triple-transgenic Alzheimer’s disease (3xTg-AD) and wild-type mice and cytokine secretion in restimulated splenocyte cultures. **a** Immunizations, blood draws, and final analyses are shown along the experimental timeline of 20 months. **b** High levels of anti-Aβ_42_ antibodies (micrograms per milliliter of plasma) were found in all of the immunized mouse groups following the last immunization (wild-type mice and 3xTg-AD mice). *Blue symbols* indicate mice that had received DNA Aβ_42_ trimer immunizations; *yellow symbols* indicate mice that had received Aβ_42_ peptide immunizations. Antibody levels of two groups of 20-month-old 3xTg-AD mice are shown as group 1 (G1) and group 2 (G2). Plasma samples had been used in a 1:1000 dilution. Samples were run in triplicates, and the assay was repeated twice. Antibody isotype analyses from DNA Aβ_42_ trimer-immunized 3xTg-AD mice (**c**) and Aβ_42_ peptide-immunized 3xTg-AD mice (**d**). *White bars* show levels of anti-Aβ_42_ antibodies of the immunoglobulin G1 (IgG1) isotype; *gray bars* show IgG2a antibody levels; *hatched bars* show IgG2b antibody levels; and *black bars* show IgM antibody levels. Differences in the amount of IgG1 (Th2) and IgG2a/c (Th1) antibody levels are statistically significant (*p* = 0.0068). Levels were measured as optical density at 450 nm (OD450). Plasma samples had been used in a 1:500 dilution, analyzed in triplicates, and the assay was repeated twice. **e** Antibody isotype profile of plasma samples from peptide-immunized mice in a 1:20,000 dilution. Interferon (IFN)-γ (**f**) and interleukin (IL)-17 (**g**) enzyme-linked immunospot analysis of splenocytes from 20-month-old 3xTg-AD mice (*n* = 4/group) and 129/SvJ wild-type mice (*n* = 4/group) that had received 13 Aβ_42_ peptide or 13 DNA Aβ_42_ trimer immunizations, respectively. No IFN-γ- or IL-17-secreting cells were found in DNA Aβ_42_-immunized mice, whereas high numbers of cells secreting IFN-γ and IL-17 were found in splenocytes from peptide-immunized mice upon Aβ_1–42_ peptide or Aβ_10–26/17–31_ peptide mix restimulation in vitro. **, and **** indicate *p* values of ≤ 0.01 and ≤ 0.001, respectively (unpaired Student's *t* test)

Immunizations were started in 4-month-old mice in groups of four to eight mice (3xTg-AD and 129/SvJ wild-type controls) with three initial immunizations in biweekly intervals (Fig. 1) with a Gal4/DNA Aβ_42_ trimer double-plasmid system (4 μg of DNA/immunization, ratio of 3:1 DNA Aβ_42_ trimer responder plasmid/Gal4 activator plasmid) via intradermal injection using a Helios gene gun (Bio-Rad Laboratories, Hercules, CA, USA) or via intraperitoneal injections of Aβ_42_ peptide (100 μg of peptide/immunization) with Quil-A (Sigma-Aldrich, St. Louis, MO, USA) as adjuvant as previously described [22–25]. The immunizations were boosted in 6-week intervals until the mice were 18 or 20 months old (up to 13 immunizations) (Fig. 1a). Control mice received Luc DNA immunizations (group 1) or no treatment (naïve controls, group 2). Blood samples were collected at different time points throughout the study 10 days following the respective immunization time points.

### IHC of mouse brains

Sagittal parallel sections of paraformaldehyde (PFA)-fixed female mouse brains were stained with antibodies specific for Aβ_42_ (6E10, BioLegend, San Diego, CA, USA; McSA1, MédiMabs, Montreal, QC, Canada; MOAB-2, MilliporeSigma, Billerica, MA, USA) to detect intraneuronal Aβ_42_ deposition and amyloid plaques in the hippocampus and cortex of the mice. To stain for tangle pathology, we used HT7, AT8, AT100, AT180, and AT270 (Thermo Fisher Scientific, Waltham, MA, USA) and T22 (MilliporeSigma); anti-tau antibodies pT231, pS214, and pS404 (Abcam, Cambridge, MA, USA); and Tyr18 (MédiMabs). NeuN antibodies (clone ABN78, MilliporeSigma; clone 1B7, Abcam) were used to stain neurons. Prior to the staining, sections were treated with heat-mediated antigen retrieval for all the tau antibodies or incubation in 70% formic acid for all the Aβ antibodies. After staining, tissues were scanned using a NanoZoomer digital pathology system and analyzed with NDP.view software (both from Hamamatsu Photonics, Shizuoka, Japan).

### Positive antibody staining area quantification

The Aβ and tau immunoreactive areas were quantified using the “area measure” tool in ImageJ software (National Institutes of Health, Bethesda, MD, USA [32]). Immunostained sections (sagittal sections of mouse brain) were imaged with a 20× objective and were converted into 8-bit grayscale. The Analyze > Measure tool was used to measure the total area occupied by positive staining in each image. The total area was averaged for the sections per mouse group. Values are arbitrary units expressed as mean ± SEM per area.

### Anti-Aβ_42_ antibody ELISA and cytokine enzyme-linked immunospot assays

ELISAs for antibody levels in mouse plasma were performed according to standard procedures. Cytokine concentrations from cell culture supernatants and enzyme-linked immunospot (ELISPOT) assays to determine frequencies of cytokine-secreting cells were performed according to standard procedures and as previously described using commercially available antibody sets for mouse interferon (IFN)-γ, interleukin (IL)-17, and IL-4 (eBioscience, San Diego, CA, USA) [23–25].

### Aβ and tau ELISAs

For semiquantitative analyses of total Aβ_42_, Aβ_40_, and tau (total tau, pT231, pS396, pT181, and pS199) levels in the brain, standard ELISAs were used (Thermo Fisher Scientific). Frozen mouse hemibrains of female mice were homogenized with a Dounce homogenizer in 10 volumes (wet brain weight) of extraction buffer [1 mM Tris, 1 mM ethylene glycol-bis(β-aminoethyl ether)-*N,N,N′,N′*-tetraacetic acid (EGTA), 1 mM dithiothreitol (DTT), 10% sucrose, pH 7.5). Following homogenization, lysates were centrifuged at 26,000 × *g* for 15 min at 4 °C to clear the homogenate. The supernatant (Sup 1) was removed, and the pellet was resuspended in 1% Triton® X-100/1 mM Tris/1 mM EGTA/1 mM DTT/10% sucrose, pH 7.5. The solution was centrifuged at 188,000 × *g* for 60 min at 4 °C. The supernatant was removed and stored at − 80 °C (detergent-soluble supernatant). The pellet was washed, dried, and dissolved in 5 M guanidine (nonsoluble fraction). Lysates containing the detergent-soluble and -nonsoluble brain fractions were further diluted in homogenate assay buffer (0.2 g/L KCl, 0.2 g/L KH_2_PO_4_, 8.0 g/L NaCl, 1.15 g/L Na_2_HPO_4_, 5% bovine serum albumin [fraction V], 0.03% Tween® 20, 1× protease inhibitor cocktail, and 1× phosphatase inhibitor cocktail, pH 7.4). Further dilutions and ELISAs were performed according to the manufacturer’s instructions.

### Western blot analysis

Soluble hemibrain lysate fractions from female mice were separated on 12% or 8–16% SDS-PAGE gels, transferred to nitrocellulose membranes (Thermo Fisher Scientific), and probed with the primary antibody overnight at 4 °C. The following antibodies were used: Tau12, 43D (BioLegend), HT7 (Thermo Fisher Scientific), anti-extracellular signal-regulated kinase 1/2 (anti-ERK1/2) and phosphorylated ERK1/2, mitogen-activated protein kinase kinase 1/2 (MEK1/2) and phosphorylated MEK1/2, GSK-3β, GSK-3α/β, glyceraldehyde 3-phosphate dehydrogenase (GAPDH), β-actin (1:1000; Cell Signaling Technology, Danvers, MA, USA), tubulin β (1:1000; Bio-Rad Laboratories), and phosphorylated glycogen synthase kinase 3β (GSK3β) Y216 (1:1000; Abcam). After incubation with horseradish peroxidase-conjugated secondary antibodies (Thermo Fisher Scientific; SouthernBiotech, Birmingham, AL, USA), antibody binding was visualized with an enhanced chemiluminescence detection reagent (ProSignal ECL; Genesee Scientific, San Diego, CA, USA) and captured on a Syngene G:Box system using GeneSys software (Syngene USA, Frederick, MD, USA). Gray-level intensities (densitometries) were quantified using gel analysis in ImageJ software [32]. For verification of similar total protein concentrations applied on the SDS-PAGE gels, filters were reprobed with housekeeping genes GAPDH, actin, and tubulin.

### Statistics

For statistical analysis (unpaired Student’s *t* test with two-tailed *p* values, nonparametric Mann-Whitney *U* test, parametric multiple comparisons one-way analysis of variance [ANOVA] and column statistics), Prism software version 6 for Windows (GraphPad Software, La Jolla, CA, USA) was used. *p* ≤ 0.05 was considered significant.

## Results

### Humoral and cellular immune responses in Aβ_42_-immunized mice

In 20-month-old transgenic mice that had received 13 immunizations (Fig. 1a), antibody levels reached 69.88 ± 11.08 μg of anti-Aβ_42_ immunoglobulin G (IgG)/ml of plasma (63.77 ± 19.53 μg anti-Aβ_42_ IgG/ml plasma in group 2) after DNA Aβ_42_ immunization and 655.9 ± 9.58 μg/ml after Aβ_42_ peptide immunization (763.4 ± 11.88 μg anti-Aβ_42_ IgG/ml plasma in group 2). Similar antibody levels were found in parallel immunized 20-month-old wild-type control animals: 49.79 ± 6.35 μg/ml in DNA Aβ_42_-immunized mice and 659.7 ± 6.95 μg/ml in Aβ_42_ peptide-immunized mice (Fig. 1b). DNA Aβ_42_ trimer-immunized mice had high levels of IgG1 and IgG2b antibodies. The overall isotype composition was IgG1 = IgG2b > IgG2a/c (IgG1/IgG2a ratio of 1.61). Low levels of IgG2a/c antibodies were consistent with a noninflammatory Th2 immune response (Fig. 1c). All peptide-immunized mice had mixed isotype profiles with similar levels of IgG1, IgG2a/c, and IgG2b antibodies, indicative of a mixed Th1/Th2 immune response (Fig. 1d). This mixed profile was found at high plasma dilutions up to 1:20,000 (Fig. 1e).

ELISPOT assays from splenocyte cultures of 3xTg-AD mice and wild-type mice were performed to detect IFN-γ (Th1 cytokine), IL-17A (Th17 cytokine), and IL-4 (Th2 cytokine) upon Aβ_42_ peptide restimulation in the immunized mice. Although we found high numbers of IFN-γ- and IL-17-secreting cells in peptide-immunized mice, low numbers of cells secreting these proinflammatory cytokines were found in DNA-immunized mice. In peptide immunized 3xTg-AD mice, IFN-γ-secreting cells were detected with 8 ± 11.27 spots in medium control wells and more than 1000 spots in the Aβ_42_ peptide restimulated wells (*p* < 0.0001 by Mann-Whitney *U* test) (Fig. 1f). We counted 104.7 ± 47.9 spots in medium control wells of DNA Aβ_42_-immunized 3xTg-AD mice with no increase in peptide restimulated wells (92 ± 39.95 spots; *p* = 0.7428). A similar pattern was observed for IL-17-secreting cells with increased numbers in Aβ_42_ peptide-immunized mice: 227 ± 15.52 spots after peptide restimulation compared with 5.3 ± 4.04 spots in medium control wells (*p* < 0.0001 by Mann Whitney *U* test) and no significant increase in IL-17-secreting cells after peptide restimulation in DNA Aβ_42_-immunized mice (87.33 ± 30.6 spots in Aβ_42_ peptide-containing wells, 111.7 ± 24.58 spots in medium control wells; *p* = 0.3439) (Fig. 1g). For a peptide mix (Aβ_10–26_/Aβ_17–31_) containing the T-cell epitope of several mouse major histocompatibility complex haplotypes (*H2*^*b*^, *H2*^*k*^, *H2*^*d*^, *H2*^*s*^), similar results were obtained in the ELISPOT assays (Fig. 1f).

### Histology showing amyloid reduction from brain

**Fig. 2:**
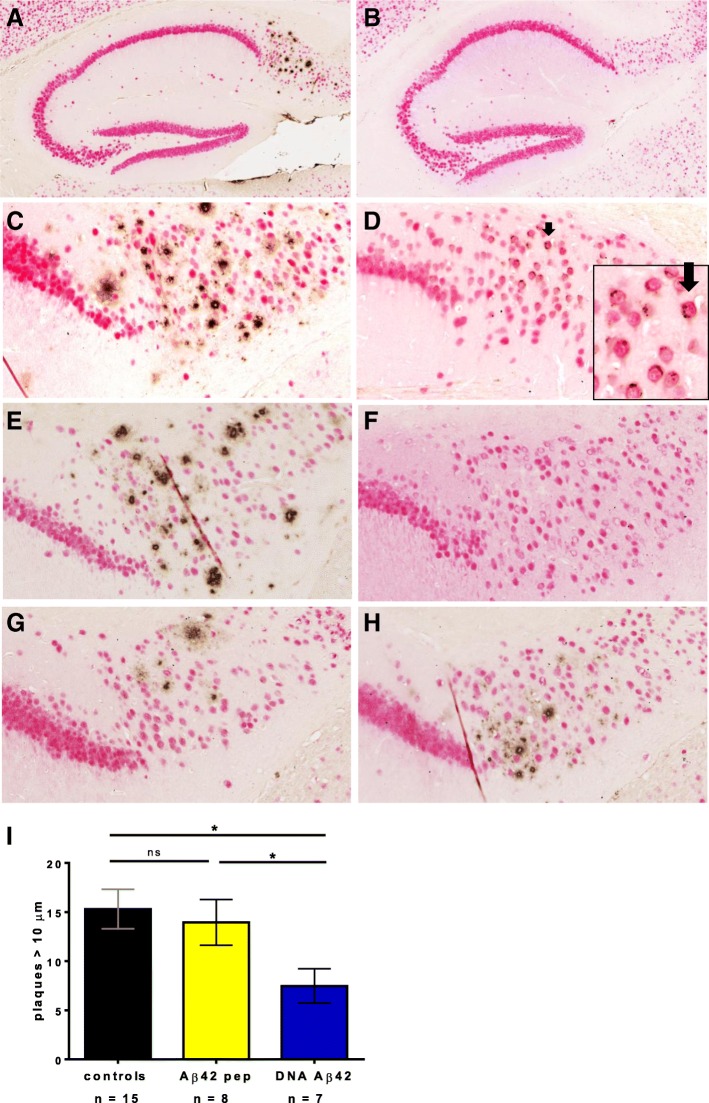
Amyloid-β (Aβ) immunization results in removal of amyloid plaques in brains of triple-transgenic Alzheimer’s disease (3xTg-AD) mice. Brain sections of mice aged 18 months (**c** and **d**) and 20 months (**a, b, e–h**) were stained with a NeuN antibody (*red*) to detect neurons and an anti-Aβ antibody (McSA1, *brown*) to detect numerous plaques in the subiculum of the hippocampus in 3xTg-AD mice. **a** The hippocampus of a 20-month-old female control 3xTg-AD mouse with numerous amyloid plaques is shown (5× magnification). **b** Hippocampus of a 20-month-old male control 3xTg-AD mouse showing no plaque pathology. **c** The subiculum of an 18-month-old female control 3xTg-AD mouse is shown at higher magnification (20×). **d** Aβ staining in the subiculum of an 18-month-old male mouse. Only intraneuronal Aβ can be detected (indicated with *arrow* and shown at higher magnification in *inset*). **e** Numerous plaques in the hippocampus of an untreated 20-month-old 3xTg-AD female mouse. **f** No plaques were found in 20-month-old wild-type mice. Both immunization regimens, Aβ_42_ peptide (**g**) and DNA Aβ_42_ (**h**), led to a reduction of plaques in 20-month-old 3xTg-AD mice compared with the control mouse (**e**). **i** Images were counted for plaques ≥ 10 μm in a 1-mm^2^ area of the subiculum/CA1 of the hippocampus by two blinded experimenters. *Blue bars* show plaque count in DNA Aβ_42_ trimer-immunized mice (*n* = 7), and *yellow bars* show plaque count found in brains of Aβ_42_ peptide-immunized mice (*n* = 8). *Black bars* show the numbers found in age- and gender-matched 3xTg-AD control mice (*n* = 15). * indicates *p* value of ≤ 0.05 (unpaired Student's t test)

In the initial studies, we used male and female mice and found large differences in the pathology between sexes. Figure 2a–d shows sections of 18- and 20-month-old mice for comparison of Aβ_42_ pathology in females and males. In 20-month-old mice, large numbers of Aβ plaques were found in the subiculum of the hippocampus in female mice (Fig. 2a), whereas no plaques were found in the 20-month-old males (Fig. 2b). Also, for tau antibody staining (HT7, AT180) in parallel sections, much less pathology was found in male mice (data not shown), and therefore we continued immunotherapy in the following groups only in females. In 18-month-old mice, amyloid plaques were abundant in the female mice (Fig. 2c). In age-matched male mice, only a few neurons with intracellular Aβ_42_ staining were found, but no plaques (Fig. 2d).

Aβ_42_ immunotherapy led to a reduction of the number of amyloid plaques in the hippocampus of treated mice. In Fig. 2e–h, staining for NeuN, which stains neurons (red color), and an Aβ antibody (McSA1), which stains amyloid plaques (brown color) are shown for the hippocampal area for representative examples of the different mouse groups in one experimental cohort. The mAb McSA1 recognizes the N-terminal region of the human Aβ peptide (Aβ_1–12_). This epitope is present in β-C-terminal fragment and amyloid precursor protein (APP) as well, but McSA1 has been reported as highly specific for Aβ as opposed to APP or soluble APP following competition studies with these antigens [33, 34]. Figure 2e shows staining of the hippocampus subiculum of a 20-month-old 3xTg-AD control female mouse. Figure 2f shows this area stained for neurons and amyloid in a 20-month-old wild-type control mouse. A reduction of amyloid plaques was seen in all mice that had received Aβ immunotherapy. Representative sections are shown for one Aβ_42_ peptide-immunized mouse (Fig. 2g) and one DNA Aβ_42_-immunized 3xTg-AD mouse (Fig. 2h).

Immunohistological staining of plaques in the brains of these mice was subjected to the counting of plaques > 10 μm in corresponding 1-mm^2^ areas (subiculum/CA1) of 15 control mice (7 DNA Aβ_42_-immunized mice and 8 Aβ_42_ peptide-immunized mice) by two blinded experimenters. These analyses showed significantly reduced plaque numbers in the DNA Aβ_42_-immunized mice (*p* = 0.0238 by Student’s *t* test compared with control mice) and a nonsignificant reduction in the Aβ_42_ peptide-immunized mice (*p* = 0.6809). Also, the difference in plaque numbers between the DNA Aβ_42_- and peptide-immunized mice was significant (*p* = 0.0487) (Fig. 2i).

### Histology showing reduction in levels of phospho-tau

**Fig. 3:**
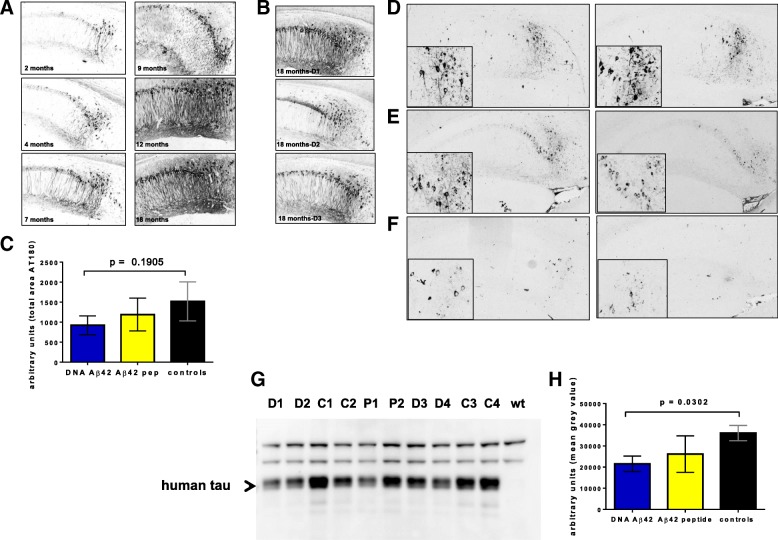
IHC staining of T231p (AT180) and T202p/S205p (AT8) in triple-transgenic Alzheimer’s disease (3xTg-AD) mouse brains and Western blots for total tau. **a** The age progression of T231p (AT180) in 3xTg-AD mouse brains is shown by IHC and staining in 2-, 4-, 7-, 9-, 12-, and 18-month-old mice. **b** T231p staining in the hippocampus of three brains from 18-month-old 3xTg-AD mice that had received DNA amyloid-β 1–42 peptide (Aβ_42_) trimer immunizations is shown for comparison. **c** Semiquantitative analyses for pT231 staining in the hippocampus of four 18-month-old control mice, five 18-month-old DNA Aβ_42_ immunized mice, six 18-month-old Aβ_42_ peptide-immunized mice, and four wild-type mice using ImageJ software (National Institutes of Health, Bethesda, MD, USA). *Blue bars* show positive areas found in DNA Aβ_42_ trimer-immunized mice, and *yellow bars* show areas found in brains of Aβ_42_ peptide-immunized mice. *Black bars* show the values of age- and gender-matched 3xTg-AD control mice. **d**–**f** DNA Aβ_42_ trimer immunization decreased AT8 staining in hippocampal sections from 20-month-old 3xTg-AD mice. **d** Representative sections from two control mice. **e** Sections from Aβ_42_ peptide-immunized mice. **f** Staining of AT8^+^ tangles in the hippocampus of two DNA Aβ_42_-immunized mice. All pictures are in 10× magnification (hippocampus); *insets* are in 40× magnification (subiculum). **g** A representative Western blot from detergent-soluble brain lysates of 20-month-old 3xTg-AD control mice (labeled C1–C4), DNA Aβ_42_-immunized mice (labeled D1–D4), Aβ_42_ peptide-immunized mice (labeled P1, P2), and a wild-type control (wt) mouse is shown. **h** Gray value intensities of human tau bands (indicated with an *arrowhead*, missing in the wt control, at 50 kD) were semiquantitatively analyzed using ImageJ software. *Black bars* show the values in 3xTg-AD control mice; *yellow bars* represent the peptide-immunized mice; and *blue bars* show values found in DNA Aβ_42_-immunized mice

The use of the 3xTg-AD mouse model allowed us to analyze a second pathology of human AD, which is the hyperphosphorylation of tau and development of neurofibrillary tangles. IHC of 3xTg-AD brain sections with different antibodies specific for tau molecules phosphorylated at specific residues (AT180, AT8, AT270, pT404, pS212, Tyr18) showed that Aβ_42_ immunotherapy also led to a significant reduction in the levels of tau phosphorylation. In Fig. 3a, the age progression for tau phosphorylation in the 3xTg-AD mouse model is shown. Brains from 2-, 4-, 7-, 9-, 12-, and 18-month-old mice (*n* = 4/group) were harvested, and PFA-fixed, paraffin-embedded sections were analyzed with the mAb AT180, which detects tau phosphorylated at residue T231. In the comparison of the staining pattern with brains from 18-month-old 3xTg-AD mice, which had received DNA Aβ_42_ immunizations, we observed that the AT180 staining intensity of the immunized 18-month-old mice appeared more like the staining intensity in brains from 7- or 9-month-old mice (Fig. 3b). Sections from four 18-month-old Luc immunized control mice, five 18-month-old DNA Aβ_42_-immunized mice, and six 18-month-old Aβ_42_ peptide-immunized mice were semiquantitatively analyzed with the area measure tool in ImageJ software. The results showed an about 40% reduction after DNA Aβ_42_ immunization and an approximately 20% reduction after Aβ_42_ peptide immunization (Fig. 3c). However, owing to high SDs and the small number of control animals, the results were not statistically significant.

Staining with the AT8 antibody specific for pS201/pT205, which is a late tau phosphorylation site [35], was less prominent in 18-month-old mice, but good staining was observed in 20-month-old mice, which showed reduction of AT8 staining in DNA Aβ_42_ trimer-immunized mice. Figure 3d shows that AT8-positive neurons were detected in the hippocampus of 20-month-old 3xTg-AD control mice (sections from two mice). Two representative sections from the Aβ_42_ peptide-immunized 20-month-old 3xTg-AD mice are shown in Fig. 3e. The brains showed fewer AT8-positive neurons than in the control animals. Much less staining was found in DNA Aβ_42_ trimer-immunized mice. Figure 3 shows the respective brain sections of the hippocampus from two mice (insets show higher magnification of subiculum in Fig. 3f).

The histological data indicating a possible reduction of tau in the Aβ_42_-immunized mice led to further substantiation of this finding by Western blot analysis and a panel of commercially available tau ELISAs that allowed testing for statistical significance of reduction of different tau phosphorylation patterns.

### Western blot analysis of total tau

**Fig. 4:**
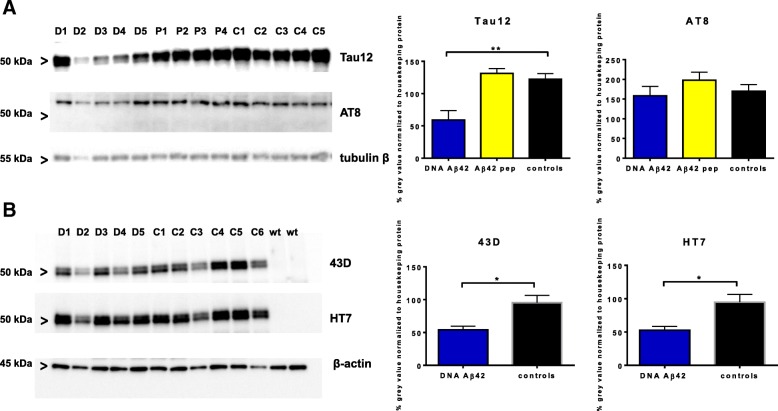
Western blot analyses for total and phosphorylated tau. Equal amounts of proteins from detergent-soluble brain lysates of 20-month-old triple-transgenic Alzheimer’s disease (3xTg-AD) mice (D1–D5 = DNA Aβ_42_-immunized mice, P1–P4 = amyloid-β 1–42 [Aβ_42_] peptide-immunized mice, C1–C5 = 3xTg-AD control mice, wt = wild-type controls) were separated by SDS-PAGE, blotted onto nitrocellulose filters, and probed using antibodies specific for total human tau (**a**, upper panel), and phosphorylated tau AT8 (**a**, middle panel), and β-tubulin as a loading control (**a**, bottom panel). The graph on the right-hand side of the SDS-PAGE pictures shows analyses of the band intensities performed with ImageJ software. All gray-level intensities of tau protein bands were normalized to the gray-level intensities of protein bands of the housekeeping proteins β-tubulin or β-actin, respectively. The reduction of total tau in the DNA-immunized mice compared with the 3xTg-AD control animals was highly significant (*p* = 0.0059). Of note, gray-level intensities for sample D2 were not included in thess calculations, because the loading control for this sample indicated a much lower protein content (**a**, bottom panel). **b** A comparison of total tau levels in DNA-immunized mice, 3xTg-AD control mice, and wt control mice in Western blots is shown using two different antibodies. In the upper panel, 43D (Tau1–100) was used for detection; in the middle panel, antibody HT7 was used; and in the lower panel, the same membrane was probed with a β-actin antibody as a protein loading control. The graph on the right-hand side of the panels shows the analyses of gray-level intensities for the protein bands with ImageJ software normalized to gray-level intensities of the housekeeping protein β-actin. Differences were statistically significant with *p* values of 0.0152 (HT7) and 0.0138 (43D). * and ** indicate *p* values of ≤ 0.05 and ≤ 0.01, respectively (Mann-Whitney* U* test)

The reduction of tau in mice that had received Aβ_42_ immunotherapy was further analyzed using Western blotting of the brain lysates. In the comparison of total tau detected with the mAb Tau12, it was found that both immunotherapies led to a reduction in tau. The reduction was not significant in Aβ_42_ peptide-immunized mice and was higher and significant in DNA Aβ_42_-immunized mice (*p* values of 0.0302, 0.0142, and 0.0023 from three independently performed Western blot analyses with detergent-soluble brain lysates). Figure 3g and h shows the results of one of these experiments (Western blot and ImageJ analysis of gray-level intensities of the bands, respectively). Total tau and phosphorylated tau were further analyzed by Western blotting, and the results are shown in Fig. 4. All band intensities were normalized to band intensities found in the reprobing of the Western blots with antibodies to housekeeping proteins. In the detergent-soluble fractions, tau detected with the mAb Tau12 was significantly reduced in brain lysates from DNA Aβ_42_-immunized mice (*p* = 0.0059 by Student’s unpaired *t* test) (Fig. 4a). The intensity of the Western blot band reactive to the mAb AT8 was only slightly reduced in DNA Aβ_42_-immunized mice (*p* = 0.3224, nonsignificant). The AT8-reactive protein band was found at higher molecular weight (about 65 kDa), which might correspond to the 64 kDa tau, a Tris-buffered saline-extractable hyperphosphorylated tau species described in the rTg4510 mouse brain (Fig. 4a, middle panel) [36]. In Fig. 4b, two different total human tau antibodies, 43D and HT7, were directly compared in parallel-run SDS-PAGE. Significant reductions of tau were found in the brain samples from DNA Aβ_42_-immunized mice (HT7 antibody, *p* = 0.0152; 43D antibody, *p* = 0.0138).

These results are consistent with the ELISA results described in the “Quantification of tau in ELISAs” section below. Although the reductions in the detergent-soluble brain lysate fractions were obvious but statistically not significant, reductions in the nonsoluble brain lysates were highly significant. However, the nonsoluble fractions could not be tested, owing to the extraction method used with 5 M guanidine for the solubilization of the pellet. These samples are not compatible with SDS-PAGE. In future mouse cohorts, we will use a different extraction protocol allowing the nonsoluble brain lysate fractions to be analyzed in Western blots (SDS-PAGE).

### Quantification of Aβ_x-42_ and Aβ_x-40_ in ELISAs

**Fig. 5:**
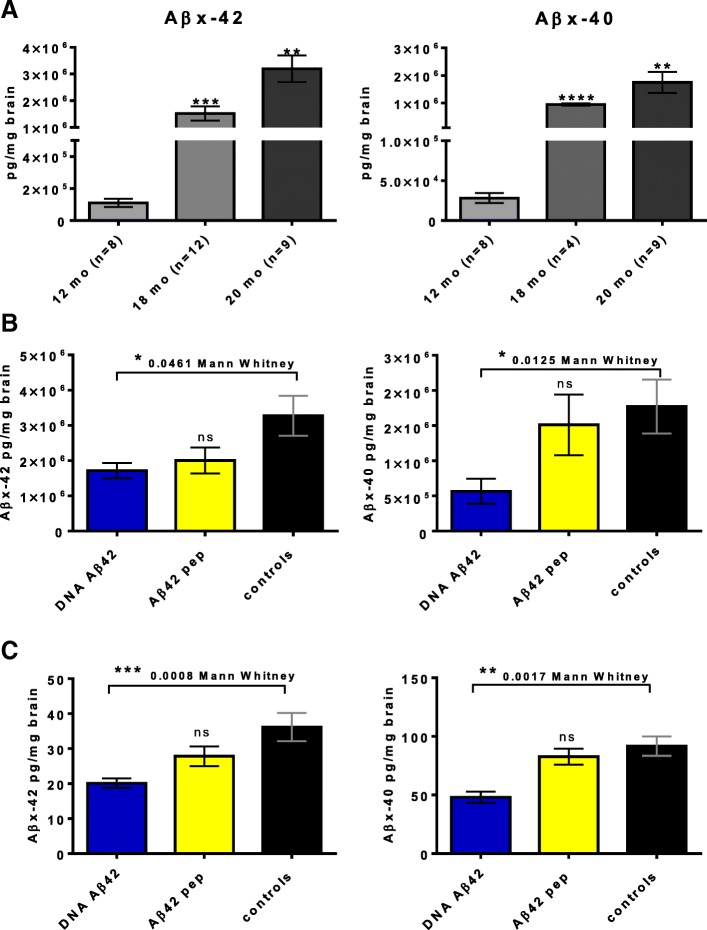
Quantitative enzyme-linked immunosorbent assay (ELISA) analyses for amyloid-β 1–42 peptide (Aβ_42_) and Aβ_40_ in brain lysates from triple-transgenic Alzheimer’s disease (3xTg-AD) mice. **a** Analyses of an increase of Aβ_42_ and Aβ_40_ peptides in brains from 3xTg-AD mice with age (12-month-, 18-month-, and 20-month-old female control mice). **b** Reduction of Aβ_42_ and Aβ_40_ peptide concentrations in the nonsoluble fractions of the brain lysates owing to Aβ_42_ immunotherapy. *Blue bars* show Aβ_42_ peptide concentrations found in brains from DNA Aβ_42_ trimer-immunized mice; *yellow bars* show the concentrations found in brains from Aβ_42_ peptide-immunized mice. The *black bars* show Aβ_42_ peptide concentrations in age- and gender-matched 3xTg-AD control mice. The left-hand graph displays data for Aβ_42_ peptides, and the right-hand graph shows data for Aβ_40_ peptides. **c** Reduction of Aβ_42_ and Aβ_40_ peptide concentrations in the soluble fractions of the brain lysates owing to Aβ_42_ immunotherapy. The left-hand graph shows data for Aβ_42_ peptides, and the right-hand graph displays data for Aβ_40_ peptides. ELISAs for the nonsoluble brain lysates were performed three times (dilution 1:10,000), and ELISAs for the detergent-soluble brain lysates were performed twice (dilution 1:2) for this particular group of mice and confirmed the data shown. * *p*  ≤0.05, ** *p* ≤ 0.01, *** *p* ≤ 0.005, and **** *p*  ≤ 0.001 (Mann-Whitney *U* test)

After analysis of brain histology as shown in Fig. 2, ELISAs were used for semiquantitative analyses of reduction of Aβ_x-40_ and Aβ_x-42_ peptides in DNA Aβ_42_ trimer- and Aβ_42_ peptide-immunized female 3xTg-AD mice. An increase of Aβ_42_ and Aβ_40_ peptides in brains from 3xTg-AD mice with age is shown in Fig. 5a. ELISAs were also used to quantify the reduction of Aβ_x-40_ and Aβ_x-42_ peptides due to DNA Aβ_42_ trimer and Aβ_42_ peptide immunization (Fig. 5b and c). Statistical significance for reduction of Aβ_42_ and Aβ_40_ was reached in the comparison of DNA Aβ_42_ trimer-immunized mice (*n* = 7, blue bars) compared with control animals (*n* = 14, black bars) in the nonsoluble fractions (*p* = 0.0461, Mann-Whitney *U* test, for Aβ_x-42_; *p* = 0.0125 for Aβ_x-40_). These reductions were nonsignificant in the one-way ANOVA (Fig. 5b). In the soluble brain lysate fractions, a reduction of both Aβ peptides was highly significant (*p* < 0.0008, Mann-Whitney *U* test; *p* = 0.0123, one-way-ANOVA, for Aβ_x-42_; *p* = 0.0017, Mann-Whitney *U* test; *p* = 0.0028, one-way ANOVA for Aβ_x-40_) (Fig. 5c) in DNA Aβ_42_-immunized mice. Aβ_x-42_ peptides were also reduced in brains from Aβ_42_ peptide-immunized 3xTg-AD mice in the nonsoluble lysate and detergent-soluble lysates, but levels did not reach statistical significance (*p* = 0.2766 for nonsoluble Aβ_x-42_, *p* = 0.0815 for soluble Aβ_x-42_). Much less removal was found for Aβ_x-40_ peptides in brains from Aβ_42_ peptide-immunized mice (Fig. 5b and c, yellow bars, right-hand graphs).

### Quantification of tau in ELISAs

**Fig. 6:**
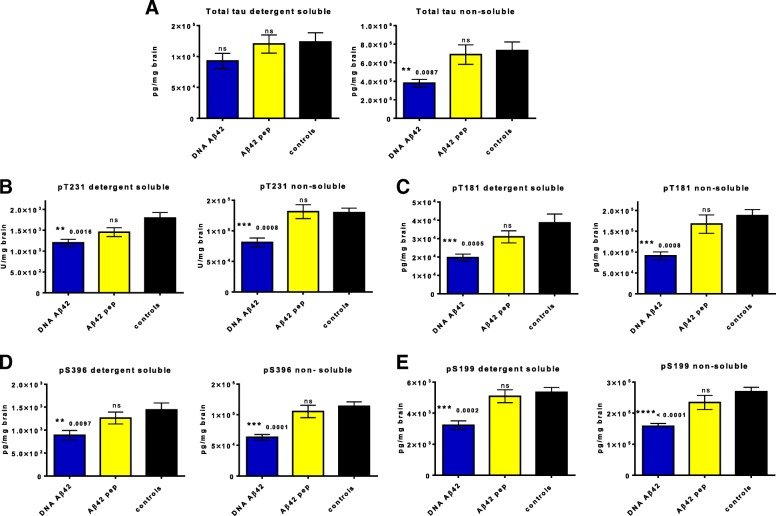
DNA amyloid-β 1–42 (Aβ_42_) immunization reduces total and phosphorylated tau in brains of triple-transgenic Alzheimer’s disease (3xTg-AD) mice. Quantitative enzyme-linked immunosorbent assay analyses for tau in detergent-soluble and nonsoluble fractions of brain lysates from 20-month-old 3xTg-AD mice. **a** Analysis of total concentrations of human tau. *Blue bars* show concentrations found in DNA Aβ_42_ trimer-immunized mice, and *yellow bars* show concentrations found in brains of Aβ_42_ peptide-immunized mice. *Black bars* show the values of age- and gender-matched 3xTg-AD control mice. The left-hand graph shows the analyses in detergent-soluble fractions from hemibrain lysates; the right-hand graph represents the analyses from nonsoluble fractions. **b** Analysis of tau phosphorylated at residue T231 (pT231), **c** Analysis of tau phosphorylated at residue S396 (pS396). **d** Analysis of tau phosphorylated at residue T181 (pT181). **e** Analysis of tau phosphorylated at residue S199 (pS199). All data are based on the analyses and comparison of 7 DNA Aβ_42_ trimer-immunized mice, 9 Aβ_42_ peptide-immunized mice, and 14 age- and gender-matched 3xTg-AD control mice. All samples were run in duplicates, and the assay was repeated twice. * *p* ≤ 0.05, ** *p*  ≤ 0.01, and *** *p*  ≤ 0.005 (Mann-Whitney *U* test)

Histological analyses of the mouse brains with tau antibodies AT180 and AT8 (early and late tau phosphorylation) showed reduced staining in the immunized mice (Fig. 3). ELISAs were used for detection of total tau, pT231 tau, p396 tau, pT181 tau, and pS199 tau in the semiquantitative analyses of tau reduction in DNA Aβ_42_ trimer- and Aβ_42_ peptide-immunized 3xTg-AD mice. Tau was reduced in both mouse groups, which had received Aβ_42_ immunotherapy or DNA or peptide vaccine (Fig. 6a–e, Table 1). However, statistical significance was reached only in the DNA Aβ_42_ trimer-immunized mice (Table 1).

**Table 1:** Tau reductions in 20-month-old female 3xTg-AD mice following Aβ42 immunotherapy

Non-soluble fraction						Detergent soluble fraction				
	DNA Aβ42 vaccine Comparison to control mice		Aβ42 pep vaccine Comparison to control mice		Comparison DNA and peptide vaccine	DNA Aβ42 vaccine Comparison to control mice		Aβ42 pep vaccine Comparison to control mice		Comparison DNA and peptide vaccine
	% tau reduction	*p* value	% tau reduction	*p* value	*p* value	% tau reduction	*p* value	% tau reduction	*p* value	*p* value
Total tau human	**- 49%**	0.0087**	- 7%	0.8446	0.0418*	**- 25%**	0.1285	- 3%	0.9754	0.2523
pT231 (AT180)	**- 37%**	0.0008***	no reduction	0.8291	0.0164*	**- 33%**	0.0016**	- 19%	0.4767	0.1142
pS396 (PHF)	**- 44%**	0.0001***	- 7%	0.4312	0.0059**	**- 39%**	0.0097**	- 12%	0.8775	0.0311*
pT181 (AT270)	**- 51%**	0.0008***	- 11%	0.5123	0.0115*	**- 49%**	0.0005***	- 20%	0.3686	0.0712
pS199	**- 42%**	< 0.0001****	− 13%	0.2496	0.0164*	**- 40%**	0.0002***	- 5%	0.5995	0.0115*

Bold letters indicate differences higher than 20% in DNA Aβ42 trimer immunized mice compared to Aβ42 peptide immunized mice; letters in bold cursive font indicate high significance for differences in reduction of tau in DNA Aβ42 trimer and Aβ42 peptide immunized mice compared to the control mice, and in the comparison of the two immunization groups (Mann Whitney u test).

* indicates *p* values ≤0.05, ** indicates *p* values ≤0.005, *** indicates *p* values ≤0.001, and **** indicates *p* values < 0.0001. All data are based on the analyses and comparison of seven DNA Aβ42 trimer immunized mice, nine Aβ42 peptide immunized mice, and 14 age and gender matched 3xTg-AD control mice

High levels of tau protein were found in the detergent soluble fractions: 1.235 × 10^5^ ± 0.556 × 10^5^ pg/ml brain lysate in the 20-month-old female 3xTg-AD control mice (*n* = 14), with small reductions in the mouse groups that had received Aβ_42_ immunotherapy (1.201 × 10^5^ ± 0.44 × 10^5^ pg/mg in the peptide-immunized mice [*p* = 0.9754, *n* = 9], and 0.928 × 10^5^ ± 0.324 × 10^5^ pg/mg [*p* = 0.1285] in DNA-immunized mice [*n* = 7]). Higher reductions of total tau were found in the nonsoluble brain lysate fractions: control mice had mean values of 7.322 × 10^5^ ± 3.301 × 10^5^ pg/mg; Aβ_42_ peptide-immunized mice had levels of 6.879 × 10^5^ ± 3.153 × 10^5^ pg/mg brain weight (*p* = 0.8446); and DNA Aβ_42_ trimer-immunized mice had significantly reduced levels of 3.793 × 10^5^ ± 1.096 × 10^6^ pg/mg brain weight of total human tau (*p* = 0.0411, one-way ANOVA) (Fig. 6a).

pT231 tau reached mean values of 1793 ± 490.3 U/mg brain weight in the detergent-soluble brain lysate fractions of 3xTg-AD control mice, 1454 ± 390.6 U/mg in Aβ_42_ peptide-immunized mice, and 1199 ± 221.5 U/mg in DNA Aβ_42_ trimer-immunized mice. Although the reduction in the peptide-immunized mice was not significant (*p* = 0.4767), the reduction in DNA-immunized mice was highly significant (*p* = 0.0091). In the nonsoluble brain fractions, a mean value of 1.296 × 10^5^ ± 0.282 × 10^5^ U/mg pT231 tau was found for control mice, 1.313 ± 0.338 × 10^5^ U/mg was found in peptide-immunized mice, and 0.809 × 10^5^ ± 0.192 × 10^5^ U/mg was found in DNA-immunized mice (Fig. 6b). Thus, Aβ immunotherapy reduced the nonsoluble pT231 only in DNA Aβ_42_ trimer-immunized mice (*p* = 0.0017).

pS396 tau was slightly reduced in the detergent-soluble fraction for DNA Aβ_42_ trimer- and Aβ_42_ peptide-immunized female mice compared with 3xTg-AD female control mice (889.2 ± 273.2 pg/mg, 1264 ± 389.1 pg/mg, and 1441 ± 566 pg/mg, respectively; *p* = ns by one-way ANOVA). pS396 was significantly reduced in DNA Aβ_42_ trimer-immunized mice in the nonsoluble fractions with mean levels of 0.631 × 10^5^ ± 0.121 × 10^5^ pg/mg (*p* = 0.0007) compared with 1.136 × 10^5^ ± 0.272 × 10^5^ pg/mg in the 3xTg-AD control mice (Fig. 6c).

For pT181 tau, mean levels of 3.869 × 10^4^± 1.774 × 10^4^ pg/mg in the detergent-soluble brain lysates of 3xTg-AD control mice were reduced to 3.098 × 10^4^ ± 0.99 × 10^4^ pg/mg in Aβ_42_ peptide-immunized mice (*p* = 0.3686) and to 1.969 × 10^4^ ± 0.507 × 10^4^ pg/mg in DNA Aβ_42_ trimer-immunized mice (*p* = 0.0198). In the nonsoluble brain fractions, a level of 1.876 × 10^5^ ± 0.591 × 10^5^ pg/mg was measured for female 3xTg-AD control mice, which was reduced to 1.672 × 10^5^ ± 0.661 × 10^5^ pg/mg (*p* = 0.5123) in Aβ_42_ peptide-immunized mice and to 0.911 × 10^5^ ± 0.248 × 10^5^ pg/mg (*p* = 0.002, one-way ANOVA) in DNA Aβ_42_ trimer-immunized 3xTg-AD mice (Fig. 6d).

pS199 tau was also reduced after DNA Aβ_42_ immunotherapy: 20-month-old 3xTg-AD control mice had a mean 5341 ± 1208 pg/mg wet brain weight in the detergent-soluble fractions, and DNA Aβ_42_ trimer-immunized mice had a reduced level of 3227 ± 730.5 pg/mg wet brain weight (*p* = 0.0012). Aβ_42_ peptide-immunized mice showed no reduction (5094 ± 1246 pg/mg, *p* = 0.5995). Significant differences were present in the nonsoluble brain lysate fractions between female control and DNA Aβ_42_ trimer-immunized mice with mean levels of 2.69 × 10^5^ ± 5.46 × 10^4^ pg/mg in control mice and 1.58 × 10^5^ ± 2.32 × 10^4^ pg/mg in DNA-immunized mice (*p* = 0.0007) (Fig. 6e, Table 1). In peptide-immunized mice the reduction was not significant, with 2.34 × 10^5^ ± 6.86 × 10^4^ pg pS199/mg (*p* = 0.2496).

In comparison of the two Aβ immunotherapies, a better reduction with high significance for phosphorylated tau molecules was found in DNA Aβ_42_ trimer-immunized mice. Percentages of reduction were calculated for the groups, and the results are shown in Table 1. A greater than 20% higher reduction was found in the detergent-soluble brain fractions of DNA-immunized mice for pT181 and pS396. This was statistically significant in the comparison of DNA- and peptide-immunized mice for pS396 (*p* < 0.0311) (Table 1). For the nonsoluble brain fractions, 12–25% higher reductions were found in lysates from DNA Aβ_42_ trimer-immunized mice for total tau, pT231, pT181, and pS396. These values were statistically significant for the comparisons with the age- and gender-matched control mice (Fig. 6) and also in the comparison between the differently immunized groups of mice (Table 1).

### Analyses of kinase variations

**Fig. 7:**
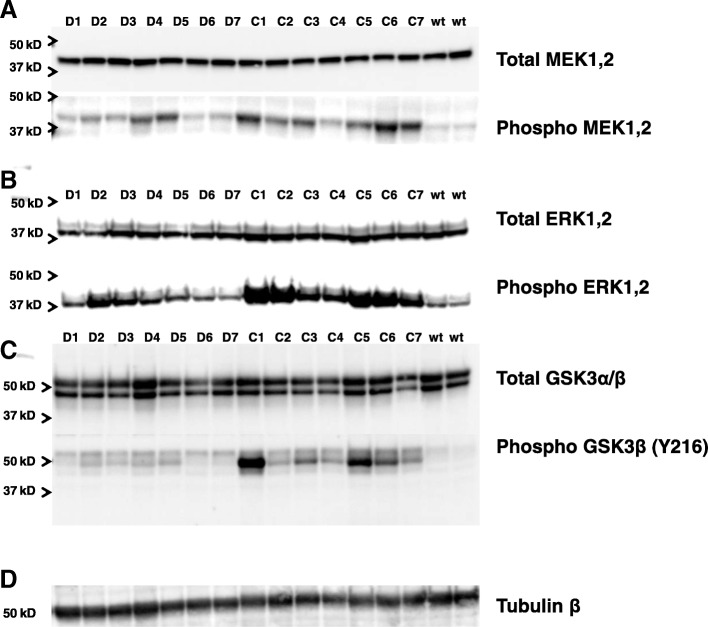
Significant changes in enzymes of the Ras/mitogen-activated protein kinase kinase (MEK)/extracellular signal-regulated kinase (ERK) signaling pathway and glycogen synthase kinase 3β (GSK3β) following DNA amyloid-β 1–42 peptide (Aβ_42_) immunization. Equal amounts of proteins from soluble brain lysates of 20-month-old triple-transgenic Alzheimer’s disease (3xTg-AD) mice (D1–D7 = DNA Aβ_42_-immunized mice, C1–C7 = 3xTg-AD control mice, wt = wild-type control mice) were separated by SDS-PAGE, blotted onto nitrocellulose filters, and probed using antibodies specific for MEK (**a**, upper panel) and its active form phosphorylated MEK (**a** lower panel), total ERK1/2 (**b**, upper panel) and the phosphorylated forms of ERK1/2 (**b** lower panel), and GSK3α/β (**c**, upper panel) and activated GSK3β (**c**, lower panel). Of note, a blot with GSK3α/β is shown in the comparison for activated GSK3β because it appears that there was weak cross-reactivity of this specific antibody with both GSK3 bands (**c**, lower panel), but differences were seen only for the strong reactivity with GSK3β phosphorylated at residue Y216 (46 kD band). As a loading control, the blots were reprobed with the housekeeping protein β-tubulin (**d**). All assays were performed three times in independent experiments. Shown are representative results from one of these assays

Western blot analyses were performed to detect whether different enzymatic kinase patterns could be found in brains from immunized mice. Significantly reduced levels of phosphorylated MEK (MAP2K), and phosphorylated ERK1/2 (p44/p42 mitogen-activated protein kinase [MAPK]), as well as reduced levels for the activated form of GSK3β (Y216), were found in brains from DNA-immunized mice. Figure 7 shows the detection MEK1/2 and phospho-MEK1/2 (Fig. 7a), as well as ERK1/2 and phosphorylated ERK1/2 (Fig. 7b), in brain lysates from seven DNA Aβ_42_-immunized mice compared with seven age- and gender-matched 3xTg-AD control mice and two 20-month-old wild-type mice.

**Fig. 8:**
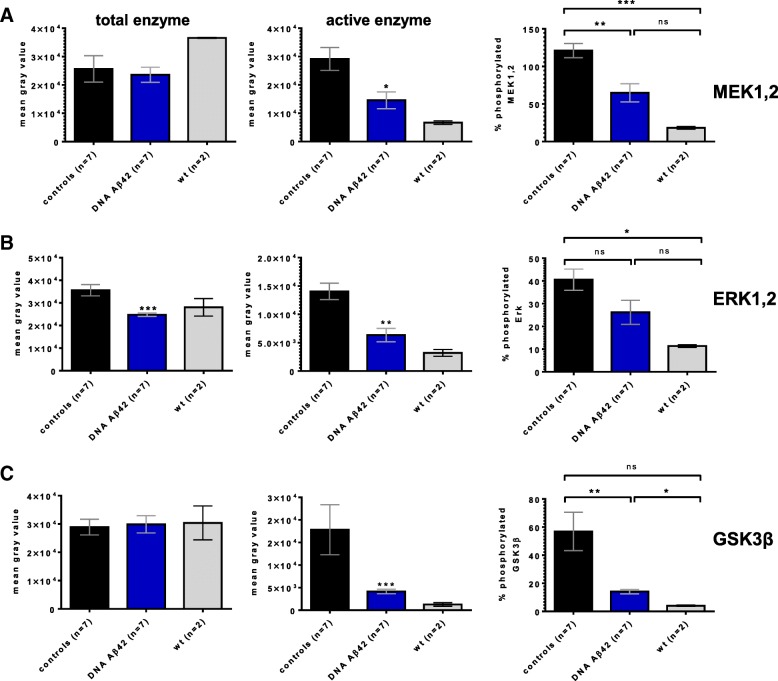
Changes in enzymes of the Ras/mitogen-activated protein kinase kinase (MEK)/extracellular signal-regulated kinase (ERK) signaling pathway and glycogen synthase kinase 3β (GSK3β) following DNA amyloid-β 1–42 peptide (Aβ_42_) immunization. Gray-level intensities (arbitrary units) of the protein bands from the Western blots shown in Fig. 7 were semiquantitatively analyzed using the ImageJ software package (National Institutes of Health). *Black bars* represent levels found in triple-transgenic Alzheimer’s disease (3xTg-AD) control mice (*n* = 7); *blue bars* represent levels found in DNA Aβ_42_ trimer-immunized mice (*n* = 7); and *gray bars* represent levels in wild-type mice (*n* = 2). **a** Analyses for MEK. **b** Analyses for ERK. **c** Analyses for GSK3β. The first graph in each row shows gray-level intensities for total enzyme; the second graph shows gray-level intensities for the active (phosphorylated) forms of the respective kinases; and the third graph shows the normalized data in which the levels of the phosphorylated kinases were calculated as a percentage of the total enzyme levels for each of the mouse brain lysates used. *, **, and *** indicate *p* values of ≤ 0.05, ≤ 0.01 and ≤ 0.005, respectively (Mann-Whitney *U* test)

Results from the semiquantitative analysis of gray-level intensities (ImageJ software) are depicted in Fig. 8. Reductions in protein levels of phospho-MEK1/2 (Fig. 8a), total ERK1/2, and phospho-ERK1/2 (Fig. 8b) in DNA Aβ_42_-immunized mice were significant (*p* values of 0.0379, 0.0006, and 0.0087, respectively, by Mann-Whitney *U* test). Significant reductions were also found for protein levels of activated GSK3β (*p* = 0.0006) (Fig. 8c). These data were further normalized against the protein levels of total MEK1/2, total ERK1/2, and total GSKα/β for each of the bands individually to compensate for the possibility of different overall protein levels for the tested enzymes in the brain lysates and shown as a percentage of protein (percentage of phosphorylated MEK, ERK, and GSKα/β). The percentage difference for phospho-MEK1/2 was highly significant between control and DNA Aβ_42_-immunized mice (*p* = 0.0031). In the comparison of phosho-ERK1/2 with total ERK1/2 in the DNA Aβ_42_-immunized mice, the reduction was not significant, because these mice already had less total ERK1/2. The percentage reduction of phospho-GSK3β (Y216) in the DNA Aβ_42_-immunized mice was highly significant (*p* = 0.006). No differences in protein levels between the mouse groups were observed for the proteins MEK1/2 (Fig. 7a), GSK3α/β (Fig. 7c), and the housekeeping protein β-tubulin (Fig. 7d). Of note, a blot with GSK3α/β is shown in the comparison for activated GSK3β because it appears that there is weak cross-reactivity of this specific antibody with both GSK3 bands (Fig. 7c, lower panel), but differences were seen only for the strong reactivity with GSK3β phosphorylated at residue Y216 (46 kD band). Only this activated form of GSK3β is described and discussed. No significant differences were found for total GSK3β protein levels in brain lysates from control and immunized mice (data not shown).

## Discussion

DNA Aβ_42_ immunotherapy results in significant reductions in Aβ_42_ peptide and plaque load in brains of the 3xTg-AD mouse model at 20 months of age, consistent with our previous results in double-transgenic mice [26, 27]. New findings shown with this vaccine for the first time were significant reductions of total tau and phosphorylated tau in brains of mice that had received active DNA Aβ_42_ trimer immunizations. This finding was confirmed by histology, Western blot analysis, and ELISA. Despite the 10× levels of anti-Aβ antibodies in peptide-immunized mice, peptide immunization was less efficacious, which is indicative of different Aβ species detected and removed by the antibodies generated following DNA Aβ_42_ immunization (e.g., Aβ oligomers). In fact, this was highly consistent throughout the study with more Aβ and more tau removed in DNA Aβ_42_ trimer-immunized mice than in Aβ_42_ peptide-immunized mice in all three assay systems used (immunohistology, Western blotting, ELISA). We had previously shown that the expression of the DNA Aβ_42_ trimer vaccine in skin shows production of Aβ oligomers [37]. We had also previously shown that the epitope specificity of antibodies produced after DNA Aβ_42_ immunization differs from the Aβ_1–15_ B-cell epitope specificity and shows a wide reactivity with epitopes across the Aβ_1–42_ peptide [38–40]. Aβ oligomers in particular activate tau kinases, leading to hyperphosphorylation, and Aβ oligomers are also strong activators for cellular caspases, leading to tau cleavage and tau aggregation. Hyperphosphorylated tau and truncated tau are both prone to self-aggregation and tau accumulation in neurons, and phosphorylation of specific residues in tau (e.g., S422) are important for caspase-mediated cleavage. Thus, less tau phosphorylation leads to less tau truncation via caspase-mediated cleavage and therefore reduces tau aggregation and total tau levels, explaining why Aβ_42_ immunization and reduction of Aβ_42_ peptides in brain led also to reduction of total tau [41–49].

DNA Aβ_42_ immunotherapy led to a noninflammatory immune response with no T-cell proliferation and no inflammatory cytokines produced during the cellular immune responses in the 3xTg-AD mouse model, similar to the immune responses we had found in the wild-type mouse model [22–25]. Although in the Balb/c wild-type mouse strain IgG1 was a dominant IgG antibody isotype in the humoral immune response [37], other mouse strains showed also a strong anti-Aβ_42_ IgG2b antibody production similar to the one found in the 3xTg-AD mouse used in the present study (unpublished data). Aβ_42_ peptide immunization led to a mixed immune response with high levels of all antibody isotypes, including IgG2a/c, and high levels of inflammatory cytokines in AD mouse models and wild-type mice [22–25, 28]. In a prime boost study, in which the immune response was first primed with Aβ_42_ peptide immunizations and then boosted with DNA Aβ_42_ immunizations in wild-type mice, we found that even though the anti-Aβ_42_ antibody isotype profile had high levels of “inflammatory” IgG2a/c antibodies, no inflammatory cytokines were detected in the cellular in vitro assays, providing evidence that the DNA immunization resulted in the downregulation of inflammatory cellular responses [50]. Antibody isotypes strongly influence the therapeutic effect of a treatment or vaccine because the different antibody isotypes have different effector functions (complement binding, Fc receptor binding). In AD immunotherapy, microglial activation is thought to help remove excess Aβ from the brain, so that FcR binding is not a negative feature of the antibody per se. Furthermore, the epitope detected by the respective antibody pool is crucial for removal of amyloid from the brain [51–53]. Thus, the multivalent nature of the humoral immune response following DNA Aβ_42_ immunization is beneficial in many aspects.

To address how either DNA Aβ_42_ or Aβ peptide vaccinations can cause both Aβ and tau reduction, we investigated a number of kinases involved in tau phosphorylation that had also been shown to be activated by Aβ_42_ peptides and in particular Aβ_42_ oligomers [54–56]. We were able to show differences for the activated/phosphorylated kinases MEK1/2, p40/p42 MAPK1 and MAPK2 (ERK1/2), and GSK-3β in brain protein lysates from female DNA Aβ_42_-immunized mice compared with the age- and gender (female)-matched 3xTg-AD control mice, supportive of the assumption that a higher removal of Aβ oligomers after DNA Aβ_42_ trimer immunization has significant effects on tau pathology via changes on cellular kinases. ERK1 and ERK2 are both highly expressed in the brain, and it had been shown in vitro that ERK2 is capable of phosphorylating a large number of residues in tau. Activation of the RAS-RAF-MEK-ERK signaling pathway by APP and Aβ_42_ oligomers in a cell culture system as well as in postmortem human AD brains indicated a pathologic link between Aβ and this particular MAPK pathway [57–59]. It has been suggested by others that the two main pathologies of AD, amyloid and tau aggregation, affect the aging brain and cause changes in large-scale neuronal circuits [60]. We show in the present study that DNA Aβ_42_ immunization led to significant changes in several pathways. Further analyses of the mechanism of action on tau reduction and changes in cellular signaling pathways in the DNA Aβ_42_ trimer-immunized mice are goals for future research.

It had been shown before in the 3xTg-AD mouse model that antitau immunotherapy or passive anti-Aβ immunotherapy led to removal of tau or Aβ or both [19, 43, 55, 61–64]. Of note, these studies were of passive immunotherapy using preformed mAbs or the intracranial injection of anti-Aβ or antitau antibodies, which is different from the active immunization done in the present study. It had also been shown before that immunization with a DNA vaccine encoding Aβ_1–11_ or a short tau epitope led to the production of antibodies against Aβ or tau, respectively [65, 66]. We show for the first time a different mechanism in which active DNA Aβ_42_ trimer immunization in the 3xTg-AD mouse model results in reduction of both pathologies with one vaccine: Aβ reduction due to antibodies generated against Aβ and tau reduction due to an indirect mechanism in which less Aβ led to less tau kinase activation and therefore to less tau phosphorylation. Of note, also in AN-1792, a clinical trial using Aβ_42_ immunotherapy in patients with AD, a trend toward reduction in cerebrospinal fluid phospho-tau concentrations was reported, and analysis of postmortem brain tissue showed a reduction of aggregated tau in neuronal processes [1–3, 67].

Current assessments of Aβ immunotherapy for the prevention of AD in several completed and ongoing trials show divergent responses [68]. Positive results in patients treated with the mAb aducanumab support the effectiveness of Aβ immunotherapy in patients with early AD [8]. Aducanumab is a fully human IgG1 antibody that corresponds to the IgG2a antibody isotype in the mouse. Of note, aducanumab has been characterized as an antibody that binds to soluble Aβ_42_ oligomers and insoluble Aβ_42_ fibrils prepared in vitro, but not Aβ_42_ monomers, consistent with the detection of conformational but not linear epitopes. This antibody reactivity might be similar to antibodies generated in response to immunization with DNA Aβ_42_ trimer as shown in the present study.

## Conclusions

We present data showing for the first time that active immunization with a DNA plasmid coding for an Aβ trimer (3xAβ_1–42_) designed to induce an anti-Aβ humoral immune response in the 3xTg-AD mouse model significantly reduced both of the main AD pathologies, amyloid and tau. We show a significant reduction in activated protein levels for p44/p44 MAPK (ERK1/2), the upstream MEK, and GSK3β. Our data support significant changes in the Ras-Raf-MEK-ERK signaling pathway in AD mouse model brain due to DNA Aβ_42_ immunotherapy, and this is a goal of further studies. DNA Aβ_42_ immunization in patients with AD has the potential to modify early and late changes in this disease. It is expected that DNA Aβ_42_ trimer immunotherapy in a clinical trial will reduce both plaques and tangles in patients with AD.

## Abbreviations

AD: Alzheimer’s disease
ANOVA: Analysis of variance
APP: Amyloid precursor protein
Aβ_42_: Amyloid-β peptide 1–42
DTT: Dithiothreitol
EGTA: Ethylene glycol-bis(β-aminoethyl ether)-*N,N,N′,N′*-tetraacetic acid
ELISA: Enzyme-linked immunosorbent assay
ELISPOT: Enzyme-linked immunospot
ERK: Extracellular signal-regulated kinase
GAPDH: Glyceraldehyde 3-phosphate dehydrogenase
GSK3β: Glycogen synthase kinase 3β
IFN: Interferon
Ig: Immunoglobulin
IL: Interleukin
mAb: Monoclonal antibody
MAPK: Mitogen-activated protein kinase
MEK: Mitogen-activated protein kinase kinase
PFA: Paraformaldehyde
